# Characterization of a Self-sufficient *Trans*-Anethole Oxygenase from *Pseudomonas putida* JYR-1

**DOI:** 10.1371/journal.pone.0073350

**Published:** 2013-09-16

**Authors:** Dongfei Han, Michael J. Sadowsky, Youhoon Chong, Hor-Gil Hur

**Affiliations:** 1 School of Environmental Science and Engineering, Gwangju Institute of Science and Technology, Gwangju, Republic of Korea; 2 Department of Soil, Water, and Climate; and BioTechnology Institute, University of Minnesota, Saint Paul, Minnesota, United States of America; 3 Department of Bioscience and Biotechnology, Konkuk University, Seoul, Republic of Korea; University of Alabama at Birmingham, United States of America

## Abstract

A novel flavoprotein monooxygenase, *trans*-anethole oxygenase (TAO), from *Pseudomonas putida* JYR-1, which is capable of catalyzing the oxidation of *trans*-anethole to *p*-anisaldehyde, was heterologously expressed in *E. coli* and purified. Enzymatic kinetics of diverse substrates and cofactors revealed that TAO is likely to be a novel self-sufficient flavoprotein monooxygenase. Enzyme assays of GST-TAO demonstrated that TAO catalyzed a *trans*-anethole oxidation reaction without auxiliary component enzyme-like electron-transfer flavin reductases. The single component TAO had the ability to reduce flavin cofactors and simultaneously oxidize *trans*-anthole to *p*-anisaldehyde. In the processes of reduction of flavin and oxidation of *trans*-anethole, TAO accepted various flavin and NAD(P)H cofactors. TAO also catalyzed oxidation of isoeugenol, *O*-methyl isoeugenol, and isosafrole, all of which contain the 2-propenyl functional group on the aromatic ring structure with different catalytic efficiency. TAO had the greatest catalytic efficiency (*k*_cat_/*K*_m_) with the original substrate, *trans*-anethole. Investigation about partially deleted mutants of TAO indicated that reductase active sites appeared to be located near the N terminal. Site directed mutagenesis studies also proved that the proposed flavin binding sites, Trp-38, Thr-43, Tyr-55, were critical for flavin reduction. However, disruption of any portion of TAO eliminated the oxygenase activity.

## Introduction

Bacterial oxygenases are important biological catalysts incorporating one or two oxygen atoms into organic compounds, which is often very difficult to perform via chemical reactions [1]. The oxygenases activate and functionalize molecular oxygen by using a reduced metal or organic cofactor through electron-transfer partners, such as iron-sulfur proteins and flavin reductases. Generally, reducing equivalents are supplied by NADH or NADPH as electron donors [2]. For example, flavoprotein monooxygenases uses a purely organic cofactor, flavin, for oxygenation reactions and utilize a general mechanisms whereby NAD(P)H reduces the flavin, and the reduced flavin reacts with O_2_ to form a C4a-(hydro) peroxyflavin intermediate, which is the oxygenation agent [3]. The oxygenation reactions catalyzed by the flavoprotein monooxygenases include hydroxylations, epoxidations, halogenations, Baeyer-Villiger oxidations, sulfoxidations, and oxidations of amines, selenide, phosphate esters and organoboron [1].

While the flavoprotein monooxygenases are classified into six groups based on sequence and structural data [1], they can also be divideded into two major classes: single-component (or self-sufficient flavoprotein monooxygenase) and a two-component enzymes composed of a reductase and an oxygenase. Hydroxylation and Baeyer-Villiger type oxidation reactions have been known to be catalyzed by either single-component or two-component flavoprotein monooxygenases [3]–[9]. However, an epoxidation reaction, which is a well known process in styrene metabolism, is catalyzed by mostly two-component flavoprotein monooxygenases [10]–[13]. It should be noted that the self-sufficient enzymes appear to have enhanced catalytic efficiency compared to the separated multi-component enzymes, which physically separate the oxygenase and reductase components. It has been postulated that the closer location between the oxygenase and reductase components in the self-sufficient enzymes results in a reduction in auto-oxidation caused by reactive oxygen species, such as hydrogen peroxide, between the two components [11], [14]. This subsequently results in an increased diffusion of the reduced FAD in the interprotein transfer process. Moreover, as expected, the self-sufficient oxygenases are better than the multi-component enzyme systems in terms of practical applications for purifying and immobilizing the enzymes in cell-free systems. For this reason, efforts have been devoted to find novel self-sufficient enzymes [15] or create artificial self-sufficient chimeric proteins [16] that have versatile activities with diverse substrates.

We previously reported [17], [18] the isolation of the *tao* gene from *Pseudomonas putida* JYR-1 encoding *trans*-anethole oxygenase (TAO) activity. The enzyme catalyzed the oxidation of *trans*-anethole, a type of phenylpropanoid compound formed via terpene biosynthesis in plants [19], to *p*-anisaldehyde. Interestingly, whole cell assays done with TAO heterologously expressed in *E. coli* showed that the enzyme also acted on isoeugenol, *O*-methyl isoeugenol, and isosafrole as substrates, all of which contain the propenyl functional group on the aromatic ring structure [18]. Compared to the extremely narrow substrate range of isoeugenol monooxygenases, Iem, from *Pseudomonas nitroreducens* Jin1 [20], and Iso from *Pseudomonas putida* IE27 [21], that only use isoeugenol as a substrate, TAO exhibited a relatively broad substrate range. TAO is likely to be NAD(P)H-dependent, even though there was no conserved NAD(P)H binding domain found from the deduced amino acid sequence [18]. Since the TAO from *Pseudomonas putida* JYR-1 displayed very low similarity to the deduced amino acid sequences of other enzymes in currently available databases, it was thought to be a novel enzyme, worthy of further characterization. In the present study, TAO tagged with glutathione *S*-transferase was heterologously expressed in *E. coli* and purified. Enzymatic kinetics of GST-TAO was investigated using diverse substrates and cofactors. Results of these studies indicated that TAO is likely a novel self-sufficient flavoprotein monooxygenase.

## Materials and Methods

### Plasmids, bacterial strains, and growth conditions

All plasmids and bacterial strains used in this study are listed in Table 1. *P. putida* JYR-1 was grown in tryptic soy broth (TSB) or Stanier's minimal salt broth (MSB) [22] containing 10 mM *trans*-anethole and incubated by rotary shaking at 200 rpm and 25°C. *E. coli* strains EPI100, EC100, DH5α [23], and BL21(DE3) were routinely grown in LB medium [24] and incubated at 37°C by rotary shaking at 200 rpm. When required, ampicillin (Amp) at 50 µg/ml, kanamycin (Kan) at 50 µg/ml, and chloramphenicol (Chl) at 12.5 µg/ml were used for selection of recombinant *E. coli*.

**Table 1:** Bacterial strains and plasmids used in this study.

Strain or plasmid	Description	Source
**Strains**		
*Pseudomonas putida* JYR-1	*trans*-Anethole transformation strain	[27]
*Escherichia coli* BL21(DE3)	Host strain for expression vector, F^−^ *omp*T *hsd*S_B_ (r_B_^−^ m_B_^−^) *gal dcm* (DE3)	Novagen
*E. coli* DH5α	Host strain for cloning vector, F^−^ *end*A1 *gln*V44 *thi*-1 *rec*A1 *rel*A1 *gyr*A96 *deo*R *nup*G φ 80d*lac*ZΔM15 Δ(*lac*ZYA-*arg*F)U169, *hsd*R17(r_K_^−^ m_K_^+^), λ^−^	[23]
**Plasmids**		
pGEX-TAO	Ap^r^; pGEX-5X-1expression vector containing *tao* gene	This study
pGEX-TAO (W38A, T43A, Y55A)	Ap^r^; pGEX-5X-1expression vector containing *tao* gene with three points mutation at Trp-38, Thr-43, and Tyr-55	This study
pGEX-TAO (N304)	Ap^r^; pGEX-5X-1expression vector containing partial *tao* gene(1–304 aa)	This study
pGEX-TAO (N261)	Ap^r^; pGEX-5X-1expression vector containing partial *tao* gene(1–261 aa)	This study
pGEX-TAO (N174)	Ap^r^; pGEX-5X-1expression vector containing partial *tao* gene(1–174 aa)	This study
pGEX-TAO (N104)	Ap^r^; pGEX-5X-1expression vector containing partial *tao* gene(1–104 aa)	This study
pGEX-TAO (C174)	Ap^r^; pGEX-5X-1expression vector containing partial *tao* gene(175–348 aa)	This study
pGEM-Teasy	Ap^r^; TA cloning vector	Promega
pG-TAO	Ap^r^; pGEM-Teasy cloning vector containing *tao* gene	This study
pTA163	Cm^r^; 41-kb pEpiFos-5 containing *tao* from JYR-1	This study

### Chemicals

*trans*-Anethole, *para*-anisaldehyde, isoeugenol, *O*-methyl isoeugenol, isosafrole, vanillin, veratraldehyde, piperonal, and 3-chloro-4-methoxybenzaldehyde were purchased from Sigma-Aldrich (Milwaukee, WI). Stock solutions (100 mM) were prepared in methanol. All organic solvents were HPLC grade and purchased from Fisher Scientific (Fair Lawn, NJ).

### Expression and purification of *trans*-anethole oxygenase

**Figure 1:**
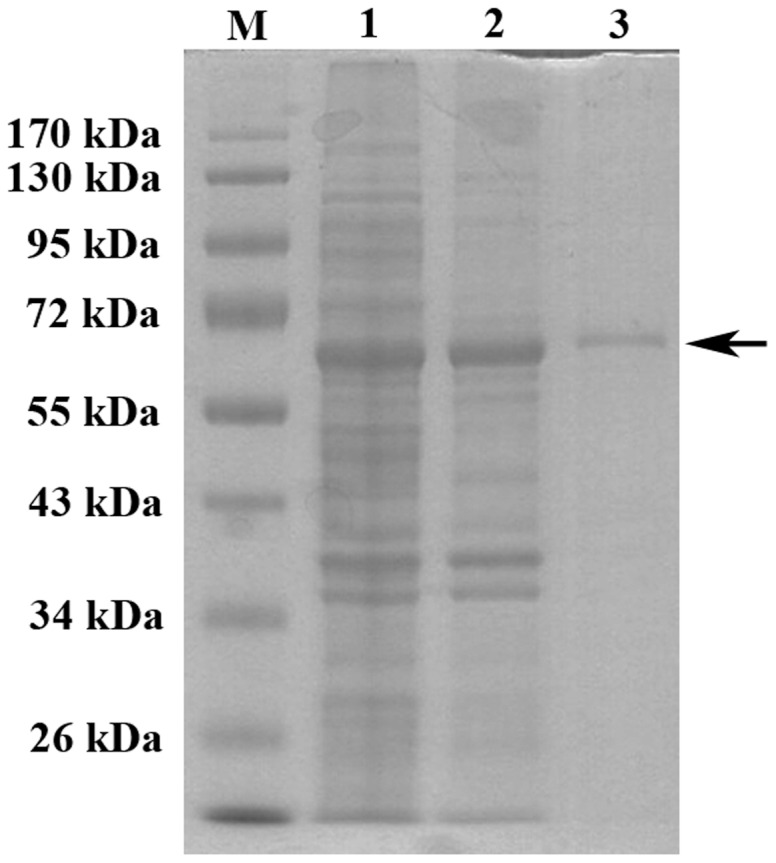
Purification of the GST-TAO. Protein samples were analyzed by SDS-PAGE at different steps of purification. Legend: M, marker protein; lane 1, cell extract; lane 2, ammonium sulfate precipitate; lane 3, elute from GSTrap column. The arrow indicates purified GST-TAO.

The full length TAO from *P. putida* JYR-1 was subcloned into the *BamH*I and *Sal*I sites of vector pGEX-5X-1 (GE Healthcare, Uppsala, Sweden), and contained glutathione *S*-transferase for the N-terminal tagging, resulting in pGEX-TAO. Expression of GST-TAO in *E. coli* BL21(DE3) (pGEX-TAO) was induced by adding 0.1 mM isopropyl-β-D-thiogalactoside (IPTG) when the culture optical density at 600 nm reached 0.5. Cells were grown for an additional 16 hr at 20°C and harvested by centrifugation at 10,000× *g* for 10 min. The cell pellet was resuspended in the PBS buffer (140 mM NaCl, 2.7 mM KCl, 10 mM Na_2_HPO_4_, 1.8 mM KH_2_PO_4_, pH 7.3) and crude cell extracts were prepared by using an ultrasonic disruptor (Cole-Parmer, Chicago, IL, USA) with 70% amplitude for 10 min (3.0 S on and 9.0 S off). The crude lysate was centrifuged, twice, at 18,000× *g* for 30 min at 4°C using PBS buffer (pH 7.3) and ammonium sulfate was added to the chilled cell extract, with stirring, to 25–35% saturation. The precipitate was collected by centrifugation at 12,000× *g* for 20 min, resuspended in PBS buffer (pH 7.3), and filtered through polyvinylidene fluoride (PVDF) syringe filters (Whatman, Maidstone, England). The filtrate was passed through a Hitrap FF desalting column connected a FPLC system (GE Healthcare, Uppsala, Sweden). The desalted elute was loaded into a GSTrap FF column (GE Healthcare, Uppsala, Sweden), which was equilibrated with 5 column volumes (CV) of PBS binding buffer, and washed with 10 CV of PBS binding buffer until no material appeared in the effluent. The GST-tagged *trans*-anethole oxygenase was eluted with elution buffer (50 mM Tris-HCl, 20 mM reduced glutathione, 10% glycerol, pH 8.8) and protein eluting at each step was applied to SDS-PAGE and visualized with Coomassie Blue staining [25] (Figure 1). The expression and purification of mutated and partially deleted TAO enzymes (Figure S1 in File S1) were processed by using the same methods.

### Determination of oxygenase activity of GST-TAO

The oxygenase activity of GST-TAO toward *trans*-anethole, isoeugenol, *O*-methyl isoeugenol, and isosafrole was measured by quantifying the corresponding aldehyde products by using high performance liquid chromatography (HPLC). The standard assay was carried out at 30°C for 1 hr, The 1 ml reaction mixture contained 200 nM GST-TAO, 10 mM NADH, 15 µM FAD, 150 mM sodium formate, 0.5 U formate dehydrogenase from *Candida boidinii* (Sigma-Aldrich, Milwaukee, WI), 20 mM Tris-HCl (pH 8.0), 1 mM *trans*-anethole, and 1 mM 3-chloro-4-methoxybenzaldehyde as internal standard. The reaction was initiated by substrate addition into reaction mixtures. Five volumes of ethyl acetate were used to extract the reaction solution. The ethyl acetate extract was evaporated in a Speed vacuum centrifugal concentrator (Vision Scientific Co., Suwon, South Korea), the residue was dissolved in 1 ml methanol, and filtered through PVDF syringe filters (Whatman, Maidstone, England). The amounts of remaining parent compounds, aldehyde product, and internal standard 3-chloro-4-methoxybenzaldehyde in the reaction solutions were determined by HPLC. Each metabolite was identified by comparison to its retention time on the HPLC column, and by UV spectrum, compared to corresponding authentic compound. Kinetic parameters of GST-TAO were obtained by using the nonlinear regression method, assuming Michaelis-Menten kinetics. The effect of pH on the activity of GST-TAO was investigated at 30°C in 100 mM of sodium acetate buffer (pH 4.0–5.8), potassium phosphate (pH 6.2–8.0), Tris-Cl buffer (pH 8.0–9.0), and glycine-NaOH buffer (pH 9.0–10.6). The effect of temperature on the activity of GST-TAO was determined by using the standard assay conditions at temperatures between 15 and 50°C. Thermal stability of GST-TAO was investigated in 20 mM Tris-HCl buffer or 20 mM phosphate buffer containing 10% glycerol and 1 mM DTT. The reaction was incubated at 25°C for 0, 12, 24, 48, 72, and 96 hr and enzyme assays were performed as described above.

### Determination of reductase activity of GST-TAO

Reductase activity of GST-TAO was determined by measuring the consumption of NADH. The standard assay contained 20 mM Tris-HCl (pH 8.0), 200 nM GST-TAO, 200 µM NADH, and 20 µM FAD in final volume of 1 ml. The reaction was initiated by the addition of NADH into the solution. Progress of the reaction was monitored, continuously, by following the decrease in absorbance of NADH at 340 nm on a UV-1601PC spectrophotometer (Shimazu Corp., Kyoto, Japan).

### Determination of FAD binding to GST-TAO and its mutants

The binding of FAD to GST-TAO and its mutants was determined by measuring the quantity of unbound FAD, which filtered out from the solution containing FAD and enzymes. Reaction solutions contained 3 µM FAD and 3 µM enzyme in 0.5 mL Tris-HCl buffer (20 mM, pH 8.0). The solution was incubated at 25°C for 60 min, loaded onto a Nanosep device centrifugal filter (Molecular Weight Cutoff (MWCO), 10K, PALL Corporation, Washington, NY) and centrifuged at 14,000× *g* for 20 min. The unbound FAD was collected and adjusted to 0.5 mL with Tris-HCl buffer (20 mM, pH 8.0). The concentration of FAD was determined by measuring fluorescence at 520 nm upon excitation at 450 nm using a Spectro-fluorometer (Spectramax Gemini XS, Gemini Scientific Corporation, Sunnyvale, CA).

### Site-directed mutagenesis

Mutations of the *tao* gene in plasmid pGEX-5X-1were introduced by using the QuikChange II Site-Directed Mutagenesis Kit (Agilent Technologies Inc., Santa Clara, CA) following the manufacturer's protocol. PCR products were digested with *Dpn*I and transformed into *E. coli* BL21(DE3) by electroporation. Transformants were selected on LB agar plates containing Amp (50 µg/ml). Plasmids from transformants were isolated using the Bionner Plasmid Mini Kit (Bionner, Daejeon, South Korea) and the desired mutations were confirmed by DNA sequencing (SolGent, Daejeon, South Korea).

### Analytical methods

Analytical HPLC was performed by using a Varian ProStar HPLC equipped with a photodiode array (PDA) detector (Varian, Walnut Creek, CA) and a reverse phase C18 column (5 µm particle size, 4.6 mm×25 cm, Waters, Milford, MA). The mobile phase, which was composed of acetonitrile containing 0.1% formic acid and water, was programmed as follows: 10% acetonitrile at 0 min, 60% acetonitrile at 10 min, 90% acetonitrile at 20 min, and 90% acetonitrile at 30 min. The injection volume was 10 µL, the flow rate was 1 mL/min, and UV detection was performed at 270 nm. LC/MS was performed by coupling an Alliance 2695 LC system (Waters Corporation, Milford, MA) to a Quattro LC triple quadrupole tandem mass spectrometer (Waters, Milford, MA) in positive electrospray ionization (ESI^+^) mode. For LC analysis, a SunFire C18 column (3.5 µm, 2.1×150 mm, Waters) was used and the mobile phase, elution program, and detection were identical to analytical HPLC described above; except the flow rate was 0.2 ml/min. For MS analysis, the source temperature, desolvation temperature, and capillary voltage were kept at 150°C, 350°C and 3.2 kV, respectively. The cone voltage was 20 V. The cone and desolvation gas were ultra-pure nitrogen at 30 and 500 L/hr, respectively. Protein concentration was determined by using the Bradford assay [26] with the Bio-Rad protein assay kit (Bio-Rad, Richmond, CA) and bovine serum albumin as a standard. All analyses were done in triplicate.

## Results

### Expression and purification of recombinant GST-TAO in *E. coli*

Due to the insolubility of recombinant TAO and His_6_-TAO in *E. coli* BL21(DE3), a GST- tagged fusion protein was selected for purifying the TAO enzyme. Recombinant GST-TAO in plasmid pGEX-TAO was found to be mainly in the soluble fraction after induction by using 0.1 mM IPTG at 20°C for 16 hours. About 23 mg of purified GST-TAO was obtained from a 2 L culture of recombinant *E. coli* cells (approximately OD_600_ = 5.0) using a series of purification steps (Table 2). The purification yield was ∼2.8% of total protein. A single, GST-TAO band of 68 kDa (calculated, 26,440 Da for GST, and 39,328 Da for TAO) was successfully observed in SDS-PAGE (Figure 1).

**Table 2:** Purification of GST-TAO from *E. coli* BL21(DE3)(pGex-TAO).

Purification step	Total volume (ml)	Concentration of protein (mg/ml)	Total protein (mg)	Total activity (units)	Specific activity (units/mg)	Purification (fold)	Yield (%)
Cell extraction	50.0	33.0	1650.0	443.3	0.27	1.0	100.0
Ammonium sulfate precipitation	37.5	4.6	172.5	83.8	0.49	1.8	18.9
GSTrap	45.0	0.5	23.4	12.6	0.54	2.0	2.8

### Kinetics and substrate specificity of GST-TAO

The catalytic kinetics of GST-TAO were determined by measuring the amount of *p*-anisaldehyde produced from the substrate *trans*-anethole [18]. The conversion of *trans*-anethole to *p*-anisaldehyde followed Mechaelis-Menton kinetics, with an affinity *K*_m_ of 64.70±2.40 µM and a turnover number *k*_cat_ of 0.49 s^−1^ (Table 3). Under the same conditions, the reaction kinetics of purified GST-TAO using isoeugenol, *O*-methyl isoeugenol, or isosafrole as substrate were determined by measuring the amount of the corresponding aldehyde product as previously reported [18]. Comparisons of the activity of GST-TAO to the four substrates indicated that TAO has the best catalytic efficiency (*k*_cat_/*K*_m_, 7.69 mM^−1^s^−1^) with *trans*-anethole as substrate. Although GST-TAO has the lowest affinity (*K*_m_, 2255.00±346.48 µM) to isoeugenol, the highest turnover number (*k*_cat_, 1.13 s^−1^) was observed with this substrate.

**Table 3:** Kinetics of GST-TAO with different substrates in the presence of NADH and FAD.

	Substrate^a^			
Parameter	*trans*-Anethole	Isoeugenol	*O*-Methyl isoeugenol	Isosafrole
*V*_max_ (units/mg)	0.23	0.52	0.06	0.26
*K*_m_ (µM)	63.70±2.40	2255.00±346.48	225.65±22.27	229.50±32.10
*k*_cat_ (s^−1^)	0.49	1.13	0.13	0.56
*k*_cat_/*K*_m_ (mM^−1^s^−1^)	7.69	0.50	0.56	2.45

a The range of substrate concentrations used in the kinetic assays is from 0.01 mM to 6 mM.

### Influence of flavin and NAD(P)H cofactors on GST-TAO activity

The activity of GST-TAO with *trans*-anethole as substrate and different flavin and NAD(P)H cofactors is shown in Table 4. Flavin and NAD(P)H cofactors were necessary for the activity of GST-TAO. When NADH was a hydride donor, the catalytic efficiency of GST-TAO with FAD (*k*_cat_/*K*_m_, 7.69 mM^−1^s^−1^) as a flavin cofactor was around two-fold greater than that of FMN (*k*_cat_/*K*_m_, 3.64 mM^−1^s^−1^) and riboflavin (*k*_cat_/*K*_m_, 3.64 mM^−1^s^−1^). The catalytic efficiency of TAO with NADH (*k*_cat_/*K*_m_, 7.69 mM^−1^s^−1^) was almost two-fold greater than that of NADPH (*k*_cat_/*K*_m_, 4.22 mM^−1^s^−1^). These results indicated that FAD and NADH were the best combined cofactors in the oxygenation of *trans*-anethole by GST-TAO (Table 4). In addition, the *K*_m_ values of GST-TAO with FAD in the presence of NADH and NADPH and *trans*-anethole as substrate were 63.7 µM and 109.5 µM, respectively. Since the *k*_cat_ values of GST-TAO to *trans*-anethole ranged from 0.41 to 0.52 regardless of the cofactor combination used for the reaction, the cofactors appear not to affect turnover numbers of GST-TAO. However, the variable *K*_m_ values with different flavin cofactors indicated that FAD is the best flavin cofactor for substrate binding. NADH was a better hydride donor for FAD reduction and the further oxygenation reaction than was NADPH.

**Table 4:** Effect of flavin and NAD(P)H cofactors on GST-TAO biotransformation activity using *trans*-anethole as substrate .

Parameters	NADH^a^			NADPH		
	FAD	FMN	Riboflavin	FAD	FMN	Riboflavin
*V*_max_ (units/mg)	0.23	0.19	0.20	0.21	0.22	0.24
*K*_m_ (µM)	63.70±2.40	112.26±21.54	120.94±39.18	109.49±2.40	160.89±11.64	270.09±37.16
*k*_cat_ (s^−1^)	0.49	0.41	0.44	0.46	0.48	0.52
*k*_cat_/*K*_m_ (mM^−1^s^−1^)	7.69	3.64	3.67	4.22	2.95	1.91

a Flavins and NAD(P)H were 15 µM and 10 mM, respectively, for saturation.

### Oxygenase and reductase activities of wildtype and partially deleted GST-TAO

**Figure 2:**
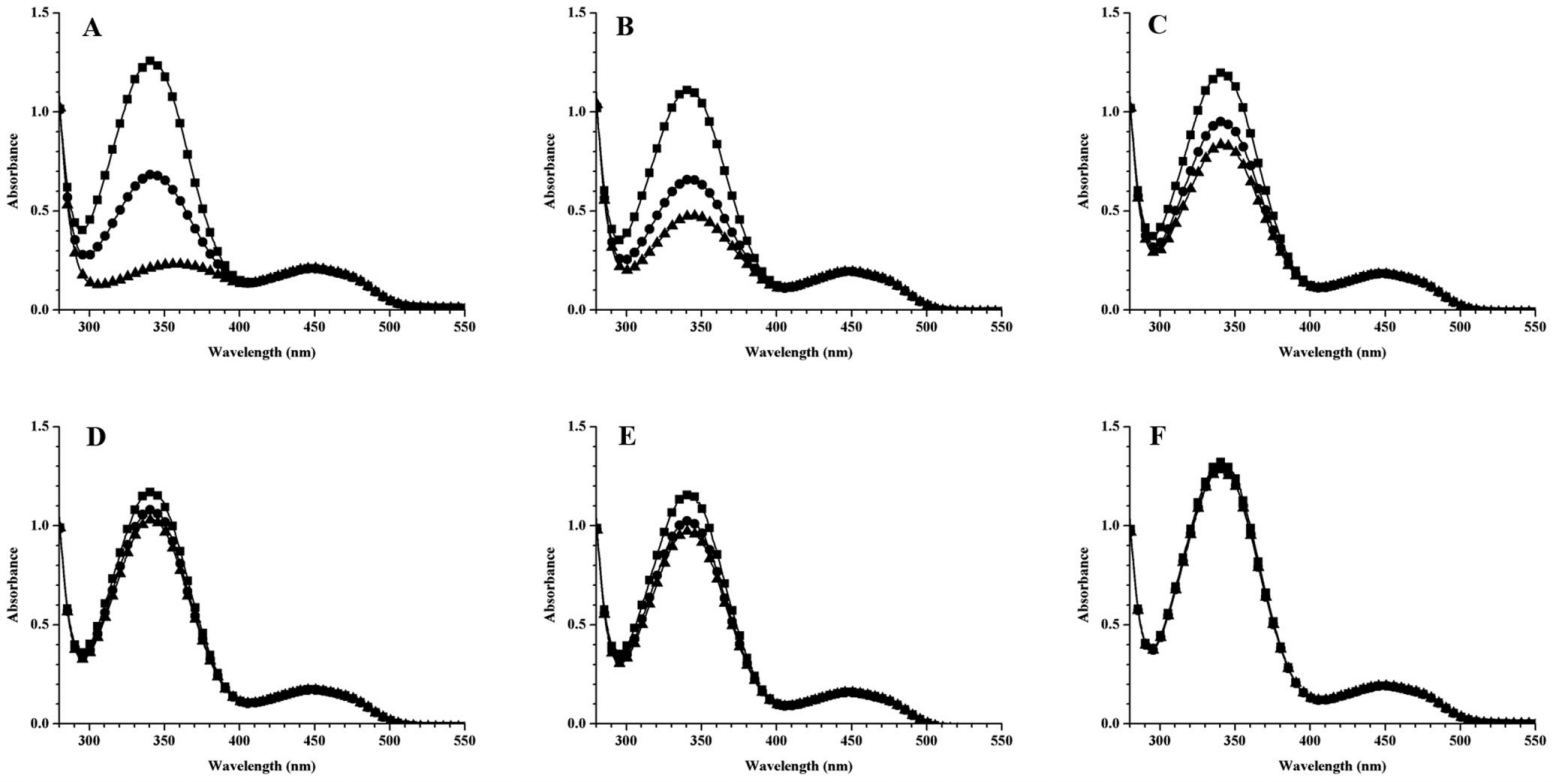
NADH consumption for reducing FAD by wild-type GST-TAO (A), its mutants GST-TAO (N1–174) (B), GST-TAO (N1–104) (C), GST-TAO (N175–348) (D), point mutated GST-TAO (W38A/T43A/Y55A) (E), and negative control (F) at 0 min (▪), 30 min (•), and 60 min (▴).

In order to profile the location of oxygenase and reductase active sites in TAO, a series of partially deleted GST-TAO mutant enzymes were expressed in *E. coli* BL21(DE3) and purified using the same method as for wild-type TAO. These mutated enzymes, which partially deleted for either their N- or C-termini, showed significantly reduced bioconversion activity (Table 5). The N-terminal GST-TAO mutants, (N1-104), (N1–174), (N1–261), and (N1–304), which partially delete the C-terminal of TAO, had relative enzyme activities of 7.3%, 6.1%, 7.6%, and 26.2% respectively (Table 5). In addition, bioconversion activity of the C-terminal GST-TAO mutant (N175–348), which partially deletes the N-terminal of TAO, was 4.7% (Table 5). A mixture of the N- and C-terminal GST-TAO mutants (N1–174), and (N175–348) did not recover the oxygenase activity. Moreover, the addition of commercial FMN-NADH reductase to the N-terminal GST-TAO mutants (N1–174), (N1–261), and (N1–304), or the C-terminal GST-TAO mutant (N175–348) also did not recover oxygenase activities (Table 5). These results indicated that the entire TAO enzyme contributed to the integrity of oxygenase activity. Figure 2 shows NADH consumptions tied to reduction of FAD by a series of the mutants and wild type GST-TAO. The N-terminal GST-TAO mutant (N1–261) consumed 123.5 µM NADH after 60 min of incubation, which is the roughly comparable amount of NADH consumed by the wild-type GST-TAO, 158.6 µM. However, the N-terminal GST-TAO mutant (N1–104), which lost more part from the C-terminal than the GST-TAO (N1–261), consumed only 50.8 µM NADH. In contrast, the C-terminal GST-TAO mutant (N175–348) lost most of its reductase activity and only consumed 12.0 µM NADH (Figure 2).

**Table 5:** Relative biotransformation activity of purified GST-TAO and its mutants in the presence of NADH and FAD.

Enzyme	Relative activity (% )^a^
Wild-type (N1–348)	100.0±2.5 (0.11±0.0 mM)^b^
GST-TAO (N1–104)	7.3±1.3
GST-TAO (N1–174)	6.1±3.0
GST-TAO (N1–261)	7.6±3.2
GST-TAO (N1–304)	26.2±1.9
GST-TAO (N175–348)	4.7±1.2
GST-TAO (N1–174)+GST-TAO (N175–348)	5.6±1.3
GST-TAO (N1–174)+Reductase^c^	4.1±3.4
GST-TAO (N1–261)+Reductase	5.1±1.9
GST-TAO (N1–304)+Reductase	28.3±7.7
GST-TAO (N175–348)+Reductase	5.9±2.1
GST-TAO (W38A/T43A/Y55A)	1.6±0.7

a Values are means ± standard deviation obtained from triplicate experiments after subtraction of the heat-killed GST-TAO activity (0.005±0.001 mM of *p*-anisaldehyde product).

b Amount of *p*-anisaldehyde produced after 60 min incubation.

c Commercial FMN-NADH oxidoreductase from *Photobacterium fischeri* was purchased from Sigma-Aldrich (Milwaukee, WI).

### FAD binding to GST-TAO and its partially deleted mutants

The binding of FAD to GST-TAO and its mutants is shown in Table 6. The N-terminal GST-TAO mutant (N1–104) still kept 67% of the FAD binding activity compared to wild-type. However, the C-terminal GST-TAO mutant (N175–348), partial deletion of the N-terminus, almost lost FAD binding activity (0.02 µmol FAD/µmol enzyme). This is about 6% of the binding activity compared to wild-type GST-TAO.

**Table 6:** Binding of FAD to purified GST-TAO and its mutants.

Enzymes	µM FAD/µM enzyme
Wild-type (N1–348)	0.30±0.05
GST-TAO (N1–104)	0.20±0.01
GST-TAO (N1–174)	0.18±0.03
GST-TAO (N1–261)	0.15±0.04
GST-TAO (N1–304)	0.19±0.04
GST-TAO (N175–348)	0.02±0.02
GST-TAO (W38A/T43A/Y55A)	0.06±0.02

### Oxygenase activitiy, reductase activitiy and FAD binding of purified targeted mutant (W38A/T43A/Y55A) of GST-TAO

Presumably due to the absence of the conserved FAD and NAD(P)H binding domains from the deduced amino acid sequence of TAO, neither the fully integrated protein structure prediction program (Prime, Schrödinger®) nor the protein structure homology modeling server (SWISS-MODEL) was able to locate the homologous protein of TAO in the protein data bank. Thus, our previous assumption [18], which the amino acid residues, Trp-38, Thr-43, and Tyr-55, in TAO are likely to be involved in FAD binding, was tested to verify the function with purified protein of the targeted mutant. Purified point-mutated GST-TAO (W38A/T43A/Y55A) lost almost all oxygenase activity (Table 5) with no consumption of NADH (Figure 2). The mutated enzyme also showed substantially decreased FAD binding activity at 0.06 µmol FAD/µmol enzyme as compared to wild-type GST-TAO at 0.30 µmol FAD/µmol enzyme (Table 6).

### Optimal reaction temperature, pH, and thermostability of TAO

The reaction temperature optimum of TAO for *trans*-anethole was determined by using the standard assay at temperatures from 15 to 50°C. The maximum activity of TAO was detected at 25°C, and an increase in temperature from 25 to 37°C resulted in loss of 90% of the activity (Figure S2A in File S1). The highest activity of TAO was observed in potassium phosphate buffer at pH 8.0 at 25°C. TAO lost about 50% of its relative bioconversion activity at pH 7.0 and 9.0 (Figure S2B in File S1).

The stability of TAO was investigated by measuring TAO activity after incubation of the enzyme at 25°C for 4 days. TAO had 76% and 30% of relative initial activity after 24 and 96 hr, respectively, suggesting that TAO is a relatively stable enzyme (Figure S3 in File S1).

## Discussion

The *trans*-anethole oxygenase (TAO) is a novel flavoprotein monooxygenase capable of catalyzing the oxidation of *trans*-anethole to *p*-anisaldehyde. The oxidation reaction was catalyzed without the aid of auxiliary oxidoreductase enzyme components. Although we observed that TAO had both monooxygenase and reductase activities, no monooxygenase or flavin reductase catalytic subunits were found in TAO. Thus, TAO is characterized as a novel self-sufficient flavoprotein monooxygenase.

**Figure 3:**
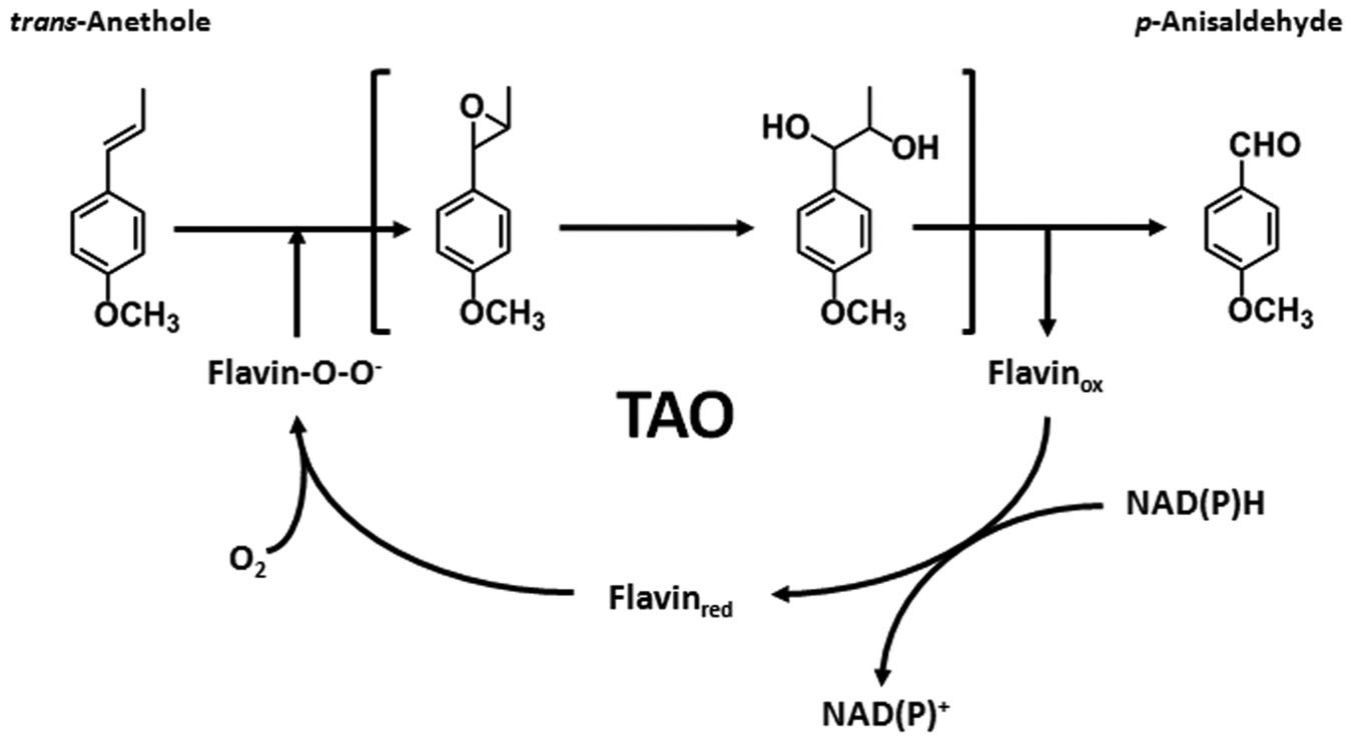
Proposed biochemical mechanism for catalyzing transformation of *trans*-anethole to *p*-anisaldehyde by TAO in the presence of NADH and FAD.

The oxidation reaction of *trans*-anethole to *p*-anisaldehyde most likely proceeded via epoxidation, hydrolysis of epoxide group, and C-C bond cleavage by TAO itself [18], [27]. TAO, which is likely a single component enzyme, not only has the ability to reduce flavin cofactors, but also oxidizes *trans*-anethole to *p*-anisaldehyde (Figure 3). Natural flavoprotein monooxygenases, which catalyze epoxidation reactions, have been previously reported as two-components monooxygenases [1]. An oxidoreductase is indispensable for the supply of reduced flavin for two-components monooxygenases. The reduced flavin diffuses to oxygenase, which reacts with molecular oxygen, and yields a reactive C4a-hydroperoxyflavin species [1], and the substrate is bound and subjected to an epoxidation reaction [14]. However, diffusion limitations likely reduce catalytic efficiency of these enzymes [12], [16]. For this reason, efforts have been made to find or construct a single-component, self-sufficient monooxygenase [14], [16]. Examples of self-sufficient monooxygenases are the *Bacillus* cytochrome P450 BM3 and P450 PFOR which integrate the entire P450 system in a single polypeptide [28], [29]. In contrast, most cytochrome P450 monooxygenases (P450s) contain multiple-components [30], [31]. Previous difficulties in finding self-sufficient monooxygenases from natural resources has led others to construct an artificial fusion between oxygenase and reductase components [16] and the catalytic activity of chimeric protein with the fusion of P450RhF and redox partner in engineered *E. coli* have been reported [32], [33]. A purified chimeric enzyme PikC cytochrome P450 fused to RhFRED showed a ∼4-fold enhanced catalytic activity (*k*_cat_/*K*_m_) in hydroxylation reaction in a macrolide biosynthetic pathway [16].

In the process of reduction of flavin and oxidation of *trans*-anethole, TAO accepted various flavins (FAD, FMN and riboflavin) and NAD(P)H cofactors serving as electron transfer intermediates. Stoichiometry indicates that flavin accepted one hydride from NAD(P)H and reduced flavin transferred one oxygen to the organic substrate, *trans*-anethole. In addition, the current study indicated that FAD is the best flavin cofactor, and NADH is better than NADPH for donating electrons to the catalytic reaction. In accordance with our initial studies done with resting cell assays of heterologously expressed TAO in *E. coli* [18], the purified GST-TAO catalyzed transformation of isoeugenol, *O*-methyl isoeugenol, and isosafrole, all of which contain a 2-propenyl functional group on the aromatic ring structure. As expected, TAO had the highest catalytic efficiency (*k*_cat_/*K*_m_) with its physiological substrate, *trans*-anethole. Isoeugenol, however, had the greatest turnover number (*k*_cat_), although the affinity for this substrate was approximately 35-fold lower than *trans*-anethole, likely due to the presence of the relatively hydrophilic 4-hydroxyl group in its chemical structure.

For profiling the distribution of the oxygenase and reductase components in TAO, partial TAO deletion mutants were constructed. Distinct difference in reductase activity between the N terminal portion of TAO, from amino acids 1–174 and the C terminal part from residues 175–348, indicated that reductase active sites appeared to be located in the N terminal half of TAO. The combined results of reductase activity (Figure 2) and FAD binding ability (Table 6) between GST-TAO (N1–174) and GST-TAO (N1–104) further indicated that the amino acids in positions 104 to 174 were critical to the FAD reduction reaction. These residues also may contribute to the binding of NADH to the enzyme. However, it should be noted that deletion of any portion of TAO resulted in loss of oxygenase activity, which is different from the reductase activity. Furthermore, the three residues Trp-38, Thr-43, and Tyr-55, which appear to be involved in FAD binding reported in our previous study [4], were confirmed to be flavin binding sites by using a FAD binding assay.

In summary, in this study we describe the identification and characterization of a novel self-sufficient *trans*-anethole oxygenase which was able to catalyze the epoxidation and cleavage of the carbon-carbon double on the 1-propenyl side chain of *trans*-anethole to produce *p*-anisaldehyde. In addition, the enzyme also coupled flavin reduction by another portion, flavin reductase. Based on the use of partial deletion mutants and point-mutations, the role of each segment in TAO for reductase and oxygenase activities could be deduced. Despite these results, however, it is necessary to further study for the catalytic mechanism of TAO by using structural analysis. We expect this single component self-sufficient *trans*-anethole oxygenase will provide another novel model system for uncovering the enzymatic function of oxygenases. Moreover, this novel enzyme has great potential as an efficient catalyst for use in the flavor and fragrance industries.

## Supporting Information

File S1 **Contans:** Figure S1. Schematic diagram of partially deleted GST-TAO. Figure S2. Optimal reaction temperature (A) and pH (B) conditions for GST-TAO activity in the presence of NADH and FAD. For the optimal pH conditions, purified GST-TAO was incubated with different buffers, 100 mM of sodium acetate buffer (pH 4.0–5.8) (▪), potassium phosphate (pH 6.2–8.0) (•), Tris-Cl buffer (pH 8.0–9.0) (▴), and glycine-NaOH buffer (pH 9.0–10.6) (▾) at 30°C for 60 min incubation time. Figure S3. Stability of GST-TAO after prolonged incubation at 25°C.
(DOC)
