# Supplementary material for: Characterization of a Self-sufficient Trans-Anethole Oxygenase from Pseudomonas putida JYR-1
Source: PLoS One. 2013 Sep 16;8(9):e73350. doi: 10.1371/journal.pone.0073350 (PMC3774712; doi:10.1371/journal.pone.0073350)

**Characterization of a Self-sufficient *trans*-Anethole Oxygenase from *Pseudomonas putida* JYR-1**

Dongfei Han^1^, Michael J. Sadowsky^2^, Youhoon Chong^3^, and Hor-Gil Hur^1*^

^1^School of Environmental Science and Engineering, Gwangju Institute of Science and Technology, Gwangju, 500-712, Republic of Korea

^2^Department of Soil, Water, and Climate; and BioTechnology Institute, University of Minnesota, St. Paul, Minnesota, 55108, USA

^3^Department of Bioscience and Biotechnology, Konkuk University, Seoul, 143-701, Republic of Korea

^*^Corresponding author

Tel: +82-62-970-2437; Fax: +82-62-970-2434; E-mail: [hghur@gist.ac.kr](mailto:hghur@gist.ac.kr)

**Supporting Information Legends**

File S1

Figure S1. Schematic diagram of partially deleted GST-TAO.

Figure S2. Optimal reaction temperature (A) and pH (B) conditions for GST-TAO activity in the presence of NADH and FAD. For the optimal pH conditions, purified GST-TAO was incubated with different buffers, 100 mM of sodium acetate buffer (pH 4.0-5.8) (■), potassium phosphate (pH 6.2-8.0) (●), Tris-Cl buffer (pH 8.0-9.0) (▲), and glycine-NaOH buffer (pH 9.0-10.6) (▼) at 30°C for 60 min incubation time.

Figure S3. Stability of GST-TAO after prolonged incubation at 25°C.

Figure S1.


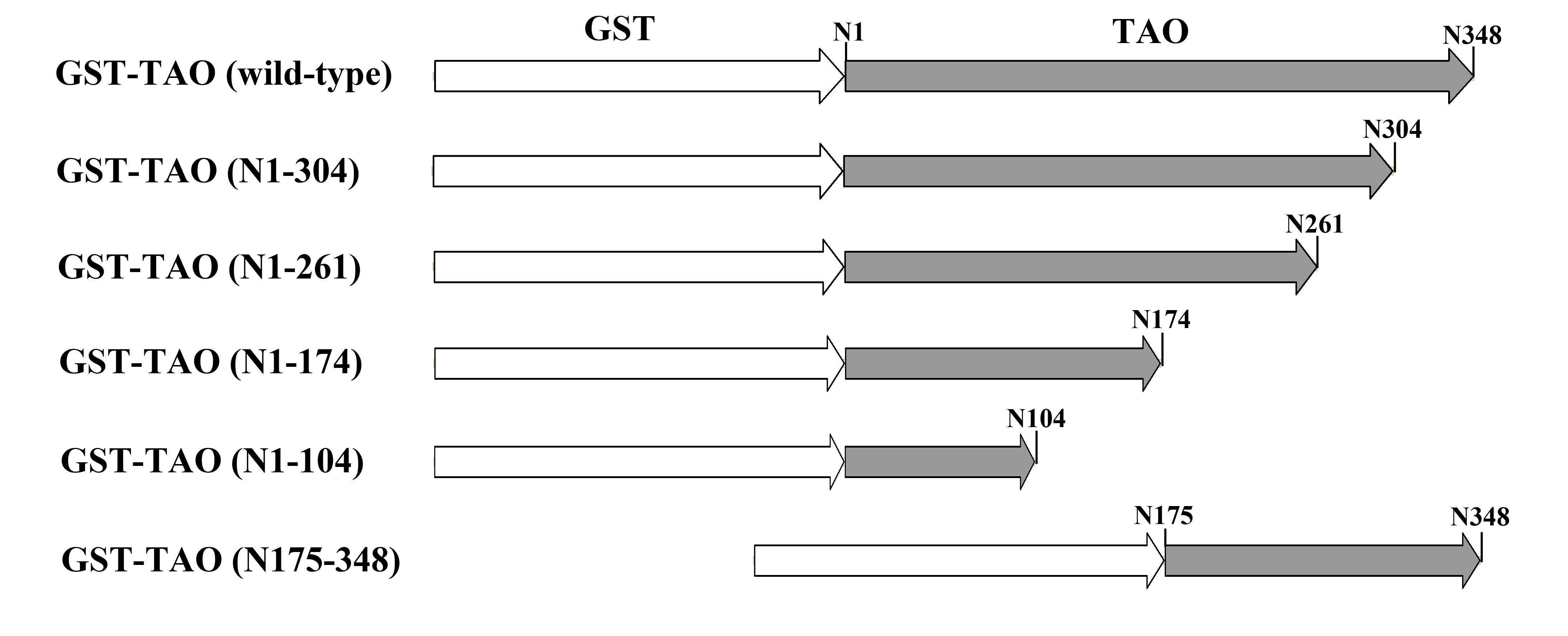


Figure S2.


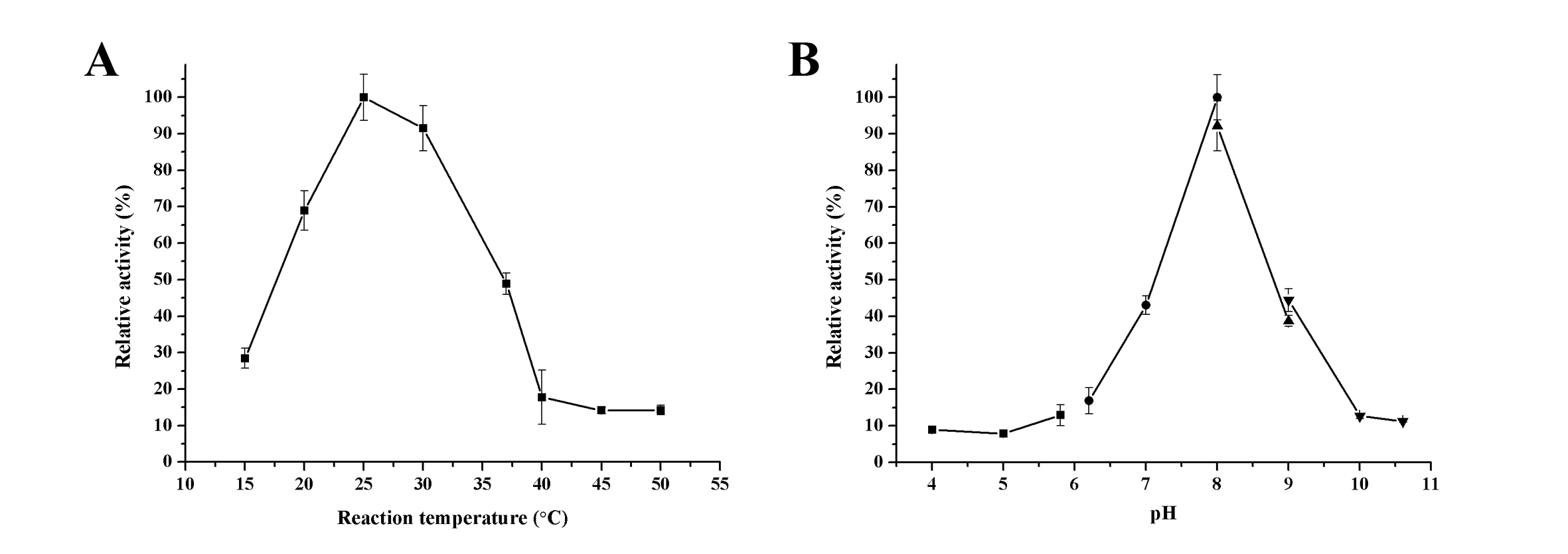


Figure S3.


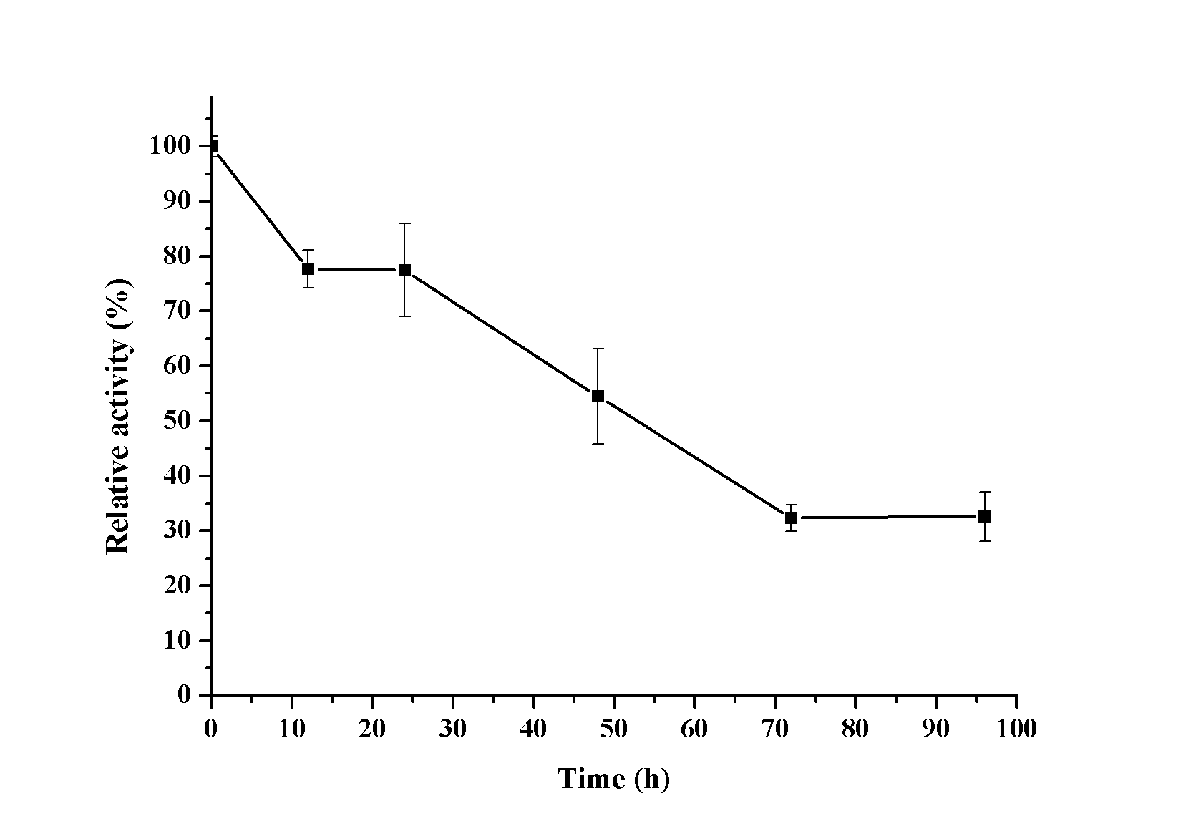

Supplement: File S1 — Contans: Figure S1. Schematic diagram of partially deleted GST-TAO. Figure S2. Optimal reaction temperature (A) and pH (B) conditions for GST-TAO activity in the presence of NADH and FAD. For the optimal pH conditions, purified GST-TAO was incubated with different buffers, 100 mM of sodium acetate buffer (pH 4.0–5.8) (▪), potassium phosphate (pH 6.2–8.0) (•), Tris-Cl buffer (pH 8.0–9.0) (▴), and glycine-NaOH buffer (pH 9.0–10.6) (▾) at 30°C for 60 min incubation time. Figure S3. Stability of GST-TAO after prolonged incubation at 25°C. (DOC) [file pone.0073350.s001.doc]
